# A Review on the Interaction of Acetic Acid Bacteria and Microbes in Food Fermentation: A Microbial Ecology Perspective

**DOI:** 10.3390/foods13162534

**Published:** 2024-08-14

**Authors:** Dong Han, Yunsong Yang, Zhantong Guo, Shuwen Dai, Mingchao Jiang, Yuanyuan Zhu, Yuqin Wang, Zhen Yu, Ke Wang, Chunchi Rong, Yongjian Yu

**Affiliations:** 1 School of Grain Science and Technology, Jiangsu University of Science and Technology, Zhenjiang 212004, China; handong@just.edu.cn (D.H.); 222241821205@stu.just.edu.cn (Z.G.);; 2 Jiangsu Provincial Engineering Research Center of Grain Bioprocessing, Jiangsu University of Science and Technology, Zhenjiang 212004, China

**Keywords:** acetic acid bacteria, fermented food, microbial interactions, *Acetobacter*, *Komagataeibacter*, *Gluconobacter*

## Abstract

In fermented foods, acetic acid bacteria (AAB), kinds of bacteria with a long history of utilization, contribute to safety, nutritional, and sensory properties primarily through acetic acid fermentation. AAB are commonly found in various fermented foods such as vinegar, sour beer, fermented cocoa and coffee beans, kefir beverages, kombucha, and sourdough. They interact and cooperate with a variety of microorganisms, resulting in the formation of diverse metabolites and the production of fermented foods with distinct flavors. Understanding the interactions between AAB and other microbes is crucial for effectively controlling and utilizing AAB in fermentation processes. However, these microbial interactions are influenced by factors such as strain type, nutritional conditions, ecological niches, and fermentation duration. In this review, we examine the relationships and research methodologies of microbial interactions and interaction studies between AAB and yeasts, lactic acid bacteria (LAB), and bacilli in different food fermentation processes involving these microorganisms. The objective of this review is to identify key interaction models involving AAB and other microorganisms. The insights gained will provide scientific guidance for the effective utilization of AAB as functional microorganisms in food fermentation processes.

## 1. Introduction

Fermented foods, characterized by the utilization of microbial growth or enzymatic catalysis to transform food raw components into distinctive textures and flavors, are nutritious food products [1]. Their multifaceted benefits include their prolonged shelf life and bolstering human immunity, improving lipid profiles, and manifesting antioxidative and anti-inflammatory properties [2]. There are often multiple types of microbes present in fermented foods [3], especially traditional fermented foods and naturally fermented foods. The ecological dynamics of microbial communities within food matrices have remained central to the discourse on food microbiology [4,5]. Fermented food ecosystems, distinct from their natural habitats, have the characteristics of being typified by batch stability [6], relatively small scales [7], limited species diversity, the culturability of most microbes, the accessibility of samples, and manipulability [8]. In the realm of food fermentation, particularly, the interactions among yeasts, LAB, AAB, and filamentous fungi orchestrate transformative processes [9]. The interaction among these microorganisms significantly influences community succession, ecological stability, and functional dynamics within fermentation ecosystems [10,11,12]. Such interactions, often mediated through molecular signaling, encompass competition, predation, microbial adaptation, and the transfer of genetic information, thereby modulating the establishment and structure of microbial consortia [12,13]. A profound understanding of microbial interactions fosters the design of synthetic microbial communities and precise control over complex fermentation processes, thereby propelling the frontier of food fermentation science.

**Figure 1:**
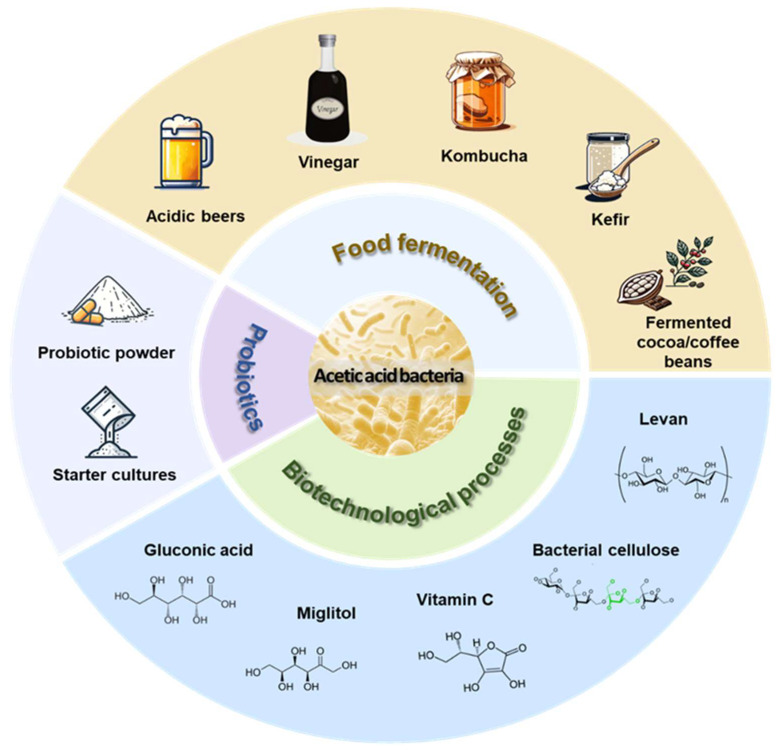
Industrial application of acetic acid bacteria.

AAB are able to incompletely oxidize sugars or alcohols into organic acids, being Gram-negative and obligately aerobic [14]. AAB proliferate ubiquitously in fruits, flowers, and naturally fermented foods, with minor occurrences in soil and insect guts [15]. Endowed with a longstanding history of safe usage, AAB hold paramount significance in industrial applications (Figure 1). However, AAB have not been as extensively investigated as many other food-grade and industrially important microorganisms. Their metabolic activities, characterized by rapid acidification through acetic acid production, in some cases produce vital compounds such as bacteriocins, extracellular polysaccharides, bacterial cellulose, aromatic compounds, and coenzymes [16,17,18]. Consequently, AAB are integral to various biotechnological processes, including the production of vitamin C, gluconic acid, miglitol, and acetic acid [19]. In the food fermentation industry, they are involved in the production of vinegar, sour beers, fermented cocoa/coffee beans, kefir beverages, kombucha, and sourdough [14,20]. In food fermentation, the main characteristic of AAB used is its ability to produce acetic acid under aerobic fermentation. The acetic acid fermentation reaction of AAB involves the oxidation of ethanol to acetaldehyde by the catalysis of pyrroloquinoline quinone-dependent alcohol dehydrogenase (PQQ-ADH), followed by the oxidation of acetaldehyde to acetic acid under the catalysis of acetaldehyde dehydrogenase (ALDH), using oxygen as the final electron acceptor and producing H_2_O_2_ or H_2_O, while generating adenosine triphosphate and releasing energy [21]. However, under unfavorable conditions (anaerobic or low oxygen concentration), alternative electron acceptors may be employed, which markedly decelerates AAB metabolism and, consequently, retards growth [22]. Recently, the probiotic properties of AAB have gained attention, highlighting their potential for development into probiotic products [23,24,25].

This review endeavors to elucidate the intricate interactions of AAB as core microbes alongside other microorganisms in food fermentation. We delineate the taxonomic classification, characteristics, and applications of AAB in fermented foods and methodologies for studying microbial interactions, and exemplify various instances of AAB interplay with other microorganisms. Through this review, we aim to elucidate the interactions between AAB and other food fermentation microorganisms in fermented foods involving AAB, and the molecular mechanisms through which these interactions occur. This information can contribute to our understanding of the mechanisms of food fermentation by complex microbial communities and provides ecological regulation references for improving the yield or optimizing the flavor of fermented foods.

## 2. The Taxonomy and Function of AAB

**Figure 2:**
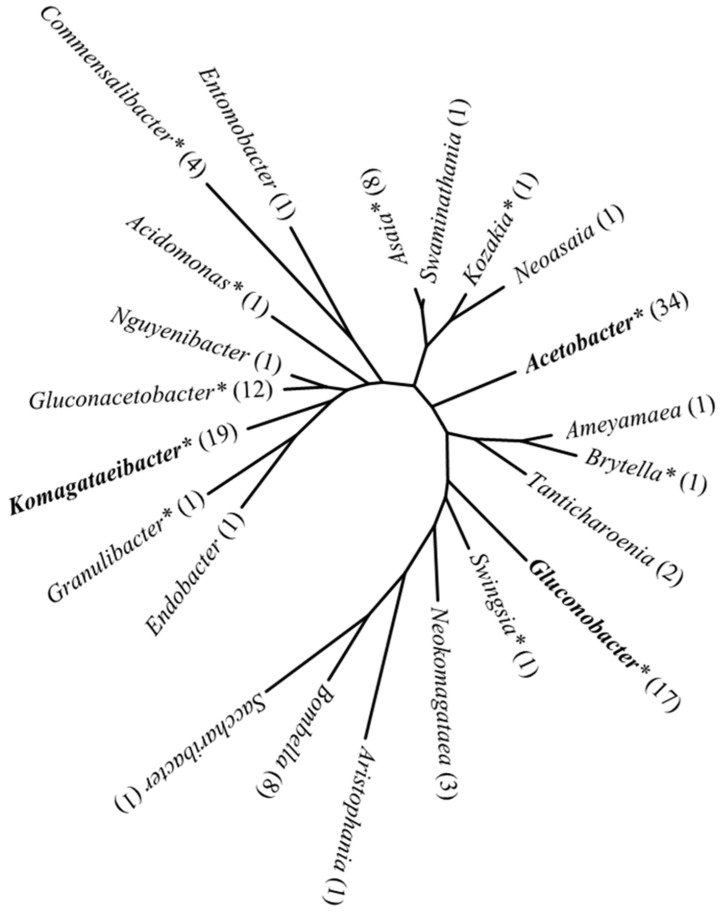
Phylogenetic tree of the AAB genus-level units based on the 16S rRNA gene. The numbers in parentheses indicate the count of validly published species within the genus. Species with more than 20 research papers using the species names as keywords in Web of Science database are marked with an asterisk, and those commonly found in food fermentation are highlighted in bold.

Since the first recognition of the genus *Acetobacter* in 1898 [26], the taxonomy of AAB has undergone significant changes [27]. Early classification systems were based on morphological and biochemical characteristics. However, with advancements in sequencing techniques, genomic-based phylogenetic analyses combined with physiology and chemotaxonomy have significantly enhanced the precision of taxonomy. Taxonomically, AAB belong to the domain *Bacteria*, phylum *Proteobacteria*, class *Alphaproteobacteria*, order *Rhodospirillales*, family *Acetobacteraceae*. *Acetobacteraceae* can be divided into two lineages, with one commonly referred to as AAB, which are phenotypically more similar. The other lineage primarily includes acidophilic bacteria, which are phenotypically more heterogeneous [28,29]. As of July 2024, AAB include 22 genera (Figure 2) and 167 species (data from List of Prokaryotic Names with Standing in Nomenclature accessed on 15 July 2024, https://lpsn.dsmz.de/) [30].

To date, the genomes of nearly all detected AAB species have been sequenced. The genome sequences of 248 strains across 21 genera have been stored in publicly accessible databases. According to EZbiocloud Genomic statistics and Genbank statistics, the smallest genomes of AAB belong to *Bombella dulcis* (1.869 Mb). The largest genomes of AAB are found in *Nguyenibacter vanlangensis* (3.923 to 4.752 Mb), *Acetobacter aceti* (3.553 to 4.877 Mb), *Acetobacter musti* (4.713 Mb), *Acetobacter sacchari* (4.553 Mb), and *Komagataeibacter intermedius* (4.465 Mb). A pan-genomic analysis of 31 strains of *Acetobacter pasteurianus* and 7 strains of *Komagataeibacter europaeus* revealed the presence of core genes, which were found to include 1181 and 2233 genes, respectively. In addition, strain-specific gene sets were identified, which consisted of 4184 and 4428 genes, respectively [31]. As new strains were added to pan-genomic analysis, the pan-genome showed a trend of gradual increase. The open pan-genome structure indicated high genetic diversity in *Acetobacter pasteurianus* and *Komagataeibacter europaeus*. Further studies are needed for comparative genomics and core-pan-genome analysis across all AAB groups.

The species frequently found in food production include *Acetobacter aceti*, *Acetobacter cerevisiae*, *Acetobacter cibinongens*, *Acetobacter ghanensis*, *Acetobacter indonesiensis*, *Acetobacter lambici*, *Acetobacter malorum*, *Acetobacter orientalis*, *Acetobacter pasteurianus*, *Acetobacter pomorum*, *Acetobacter senegalensis*, *Gluconobacter cerevisiae*, *Gluconobacter oxydans*, *Komagataeibacter europaeus*, *Komagataeibacter hansenii*, and *Komagataeibacter xylinus* (Table 1). Among these, *Acetobacter pasteurianus*, *Komagataeibacter europaeus,* and *Gluconobacter oxydans* are usually the most dominant species in food applications.

*Acetobacter pasteurianus* is a common microbe in acidic fermented foods and a key AAB in vinegar fermentation. *Acetobacter pasteurianus* efficiently converts ethanol into acetic acid via ethanol dehydrogenase and aldehyde dehydrogenase. It typically exhibits a high rate of acetic acid production and good tolerance (~6% *w*/*v*), with a final acetic acid concentration generally not exceeding 8% (*w*/*v*) [67]. Hence, it is widely used in solid-state grain vinegars and surface fermentation vinegars, particularly in Asian and Europe. It is initially used as a rapid fermentation startup in liquid-submerged vinegar fermentation but is often replaced by *Komagataeibacter europaeus* in the later stages due to its inability to tolerate higher acetic acid concentrations. Strains of *Acetobacter pasteurianus*, with more than 280 transposons and some genes containing hypermutated tandem repeats and several plasmids in their genomes, exhibit physiological instability [68]. That is why AAB from older tanks rather than preserved pure cultures are commonly used for inoculation in industrial production. Another disadvantage is that, in the absence of ethanol, acetic acid can be further oxidized to CO_2_ via the tricarboxylic acid (TCA) cycle. This leads to so-called overoxidation of acetate, which results in a reduction in the fermentation product. To prevent overoxidation, an oxygen-excluding method is employed once a peak acetic acid accumulation is detected, causing a rapid decline in the viable cell count. A comparative genomic analysis of 13 *Acetobacter pasteurianus* strains revealed rearrangements in gene distribution, but the ADHs and ALDHs responsible for acetic acid production were highly conserved [69]. Recent studies on the probiotic properties of *Acetobacter pasteurianus* found that strain BP2201 possesses strong acid and bile salt tolerance, efficient ethanol degradation, potent antioxidative activity, and sensitivity to specific antibiotics [23], with the potential to lower blood lipid levels, alleviate oxidative stress, improve alcoholic liver damage, and modulate gut microbiota, thus mitigating alcohol-induced diseases [24].

*Komagataeibacter europaeus* (originally named *Gluconoacetobacter europaeus* or *Acetobacter europaeus*) was first observed by Sievers in 1992 and characterized from high-acid vinegar fermentations in Central Europe [70]. This species, due to its unique growth characteristics, has become one of the most suitable AAB for industrial vinegar production. These characteristics include a high capacity for oxidizing ethanol and producing acetic acid, with a good tolerance to ethanol (0–20% *w*/*v*) and acetic acid (15–20% *w*/*v*) [71]. *Komagataeibacter europaeus* can thrive in environments with pH as low as 2.5, but requires a continuous supply of oxygen for growth [72]. These metabolic features make *Komagataeibacter europaeus* particularly suitable for growth in various vinegars produced by submerged cultivation, commonly found in spirit, white wine, and red wine vinegars. However, other AAB species may also be present in various vinegars. Moreover, when a mixed starter culture of AAB is used in submerged vinegar production, *Komagataeibacter europaeus* tends to dominate in the late fermentation stage [72]. PQQ-dependent ADH activity in *Komagataeibacter europaeus* is twice that of *Acetobacter pasteurianus*. Under the presence of acetic acid, the purified enzyme activity of *Acetobacter pasteurianus* declines faster than that of *Komagataeibacter europaeus* [21]. This shift in dominance highlights the competitive advantage of *Komagataeibacter europaeus* in environments rich in acetic acid. *Komagataeibacter europaeus* can also be used for the production of bacterial nanocellulose [73], and certain species within the *Komagataeibacter* genus possess the capability to produce bacterial cellulose, such as *Komagataeibacter xylinus*, *Komagataeibacter hansenii*, and *Komagataeibacter nataicola* [74,75,76]. The produced bacterial cellulose can be applied in food ingredients (such as desserts and artificial meats), food packaging materials (such as casings for sausages and meats), and as additives in low-calorie foods (acting as thickeners, suspending agents, and stabilizers), as well as in delivery systems, enzymes, and cell immobilization [77].

*Gluconobacter oxydans* was first identified and described in the early 20th century, drawing scientific attention due to its potent oxidative abilities. Its natural distribution mirrors that of common AAB, being found in soil, fruits, sake, and fruit wines, as well as in nectar and pollen [78,79]. This organism exhibits a strong capability to convert various sugars, polyols, and alcohols into acids, aldehydes, and ketones [80]. However, *Gluconobacter oxydans* produces acetic acid at a slower rate than *Acetobacter* and does not oxidize acetic acid. In submerged fermentation, *Gluconobacter oxydans* produces a large amount of gluconic acid in addition to acetic acid, so it is not widely used in the vinegar industry [81]. However, *Gluconobacter oxydans* is extensively utilized in the biotechnology sector for the fermentation production of L-ascorbic acid (vitamin C), miglitol, gluconic acid and its derivatives, and dihydroxyacetone [82]. It is also employed in other areas such as the preparation of biosensors [79].

## 3. Types of Microbial Interactions

**Figure 3:**
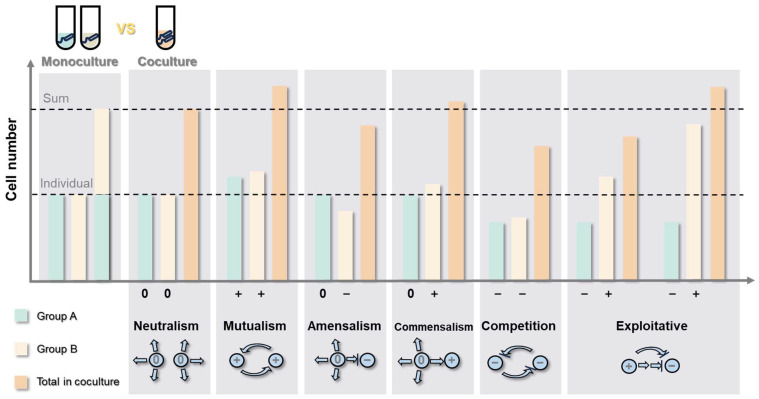
The model for classifying interactions between two species or strains in monoculture and co-culture.

In fermented food environments, multiple microorganisms often participate, forming complex interaction networks. These interactions within the network can positively impact species, negatively affect them, or have no interference. Based on the effects on paired species, several primary interaction types can be identified: neutralism, mutualism, amensalism, commensalism, competition, and exploitative (Figure 3).

Symbiotic relationships include mutualism and protocooperation. In fermented foods, interactions between microorganisms are beneficial to both parties but are not essential. That is, the two strains can continue to grow independently after separation and are considered mutualistic. For instance, the mutually beneficial symbiosis between *Streptococcus thermophilus* and *Lactobacillus delbrueckii* subsp. *bulgaricus* in yogurt cultures positively affects both their growth rate and population size [83]. During sourdough fermentation, LAB and yeast co-culture enhances each other’s growth; *Fructilactobacillus sanfranciscensis* is stimulated by non-protein factors and CO_2_ produced by *Saccharomyces cerevisiae*, while *Saccharomyces cerevisiae* thrives on acids produced by the LAB [84]. Mutualistic relationships are widespread in nature. Biofilms are a typical example of mutualism, consisting of microbial aggregates and adhesions forming film-like or granular structures (kefir grains, kombucha). The biofilm lifestyle is advantageous for microbial adaptation in harsh environments. But members within a biofilm not only engage in mutualism but also experience competition and other interactions [85].

Amensalism is an interaction where one party is harmed while the other remains unaffected. This relationship is frequently employed in food fermentation to inhibit the growth of spoilage microorganisms. Ethanol is produced by yeast, while lactic acid is produced by LAB, both of which serve to create the desired fermented food. Another example in traditional solid-state fermentation, bacilli srtains isolated from Daqu are used to inhibit the main producers of geosmin, *Streptomyces sampsonii*, effectively eliminating harmful flavors in Baijiu using biological antagonism [86].

In commensalism, one party benefits while the other is not significantly affected. In the fermentation of wine, the relationship between *Saccharomyces cerevisiae* and LAB often manifests as commensalism. The co-inoculation of red wine with *Saccharomyces cerevisiae* and *Oenococcus oeni* for malolactic fermentation benefits the growth of the yeast, while the LAB has no significant effect on the yeast. This synergistic interaction can shorten the fermentation period [87]. In caproic acid production, a binary biological system involving *Clostridium kluyveri* H068 and *Methanogen* 166 is constructed. The *Methanogen* can convert hydrogen produced by *Clostridium kluyveri* into methane, relieving hydrogen-mediated feedback inhibition and thus enhancing caproic acid production. This interaction mechanism can be applied to improve the content of hexanoic acid ethyl ester, a crucial flavor component, in Chinese strong-aroma-type baijiu [88].

Competition refers to two interacting parties competing for nutrients or space. This type of interaction typically has detrimental effects on both species, but its impact on the community as a whole requires further study. Competition for nutrients is evident in various fermentations. The competition for carbon and nitrogen sources in common food fermentations plays a crucial role in determining microbial succession and the accumulation of fermentation products. In the fermentation of Chinese rice wine, the accumulation of biogenic amines is promoted through the cross-feeding of amino acids among amine-producing LAB. By competing with amine-producing LAB, *Lactiplantibacillus plantarum* (ACBC271) disrupts their activity, reducing the total biogenic amine content by 22% (not exceeding 43.54 mg/L), thus decreasing the accumulation of harmful components in fermented foods [89].

Exploitative relationships include parasitic and predatory relationships. Among microorganisms, they are similar, in that one organism is suppressed (consumed), while the other benefits. Phages are typical parasites and pose significant risks in fermentation industries, especially when the same equipment is reused, making them susceptible to phage attacks. A phage attack can deactivate dominant strains in the fermentation medium, leading to industrial fermentation failures and product losses. Natural predatory bacteria also exist, which can parasitize and kill bacterial hosts. Using naturally occurring *Vibrio* predatory bacteria to control pathogenic vibrios in seawater and shellfish is significant. When pathogenic *Vibrio parahaemolyticus* O3:K6 is introduced into natural seawater containing detectable amounts of predatory vibrios, the pathogen’s numbers reduce to undetectable levels within 48 h [90].

The relationships among microorganisms are not static. The same microbes can exhibit different interactions depending on the environment or over time. Research based on four microbial strains exhibited more positive interactions when using a lubricant as the sole carbon source. However, when other nutrients and detoxifying agents were added to the culture system, the relationship among the four strains shifted from facilitation to competition. This research reveals that harsh environments promote microbial cooperation, whereas rich environments more commonly foster competition [91]. Another study, employing rigorous and extensive co-culture experiments (3008 bacterial pairs), found that mutualism relationships comprised 39.1% of the total, while competitive relationships accounted for only 13.9%. The study revealed that mutualism relationships between bacteria are significantly enhanced under conditions of high temperatures and extreme stress. That is, “difficult environments promote microbial cooperation” [92]. The preceding study indicates that when studying the interactions between microorganisms, it is essential to consider the environment in which these microorganisms are situated.

Time scale is also a factor affecting microbial interactions, reflecting changes in the environment caused by the microbes themselves. Studies show that microbial interactions dynamically change over time in a batch culture system. The two marine bacteria initially engage in positive interactions, but later shift to competitive relationships due to environmental changes caused by the metabolites they secrete [93].

## 4. Methods for Studying Microbial Interactions

### 4.1. Top-Down Approach

**Figure 4:**
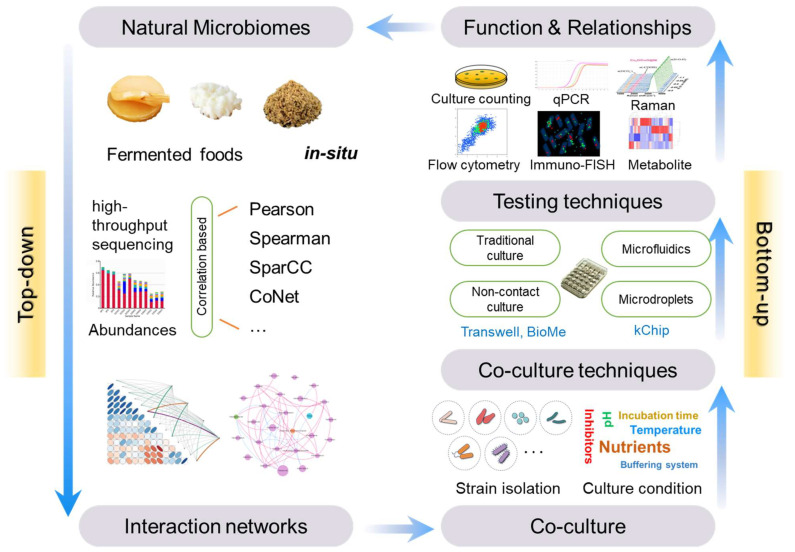
Methods for studying microbial interactions in fermented foods.

Microbial interaction networks are commonly used in ecology to decipher complex patterns of microbial interactions. These interactions can be analyzed from non-culture-dependent microbiome data, which are more reflective of in situ conditions and avoid alterations caused by culturing conditions. Initially, high-throughput sequencing data obtained in situ are used to calculate correlation coefficients between nodes (representing taxonomic groups at different classification levels), such as the Pearson, Spearman, SparCC, and CoNet interaction estimates [94]. Typically, these networks are undirected. The existing microbial ecological networks struggle to differentiate between direct and indirect effects and are affected by compositionality bias, overdispersion, poor sample-to-feature ratios, and trans-kingdom interactions [95]. More reliable results require experimental studies of microbial communities for validation. These methods (Figure 4) have been applied in the study of microbial ecology in the human microbiome [94], natural environment [96,97], and fermented foods [98,99].

### 4.2. Bottom-Up Approach

The analysis of microbial interactions in the bottom-up approach primarily involves co-culture techniques and an assessment of microbial phenotypes (Figure 4). Common microbial phenotype assessments include microscopy, electron microscopy, culture-dependent methods, and quantitative PCR for measuring the biomass difference, GC-MS for analyzing changes in primary metabolites, flow cytometry, immunofluorescence, Raman spectroscopy, phospholipid fatty acid (PLFA) analysis, and fluorescence in situ hybridization (FISH). The gold standard for identifying interactions between two microbial strains using culturing methods was summarized by Kevin’s team [100]. This standard involves comparing the productivity of two microbial strains in co-culture versus individual cultures, typically measured by cell numbers or biomass. The results are categorized into 11 different scenarios, defining six types of interaction relationships (Figure 3).

Additionally, to increase the throughput of microbial interaction studies, microfluidic, microdroplet, and microchamber culturing technologies are also employed. Microfluidic technologies can construct micro-bioreactors, allowing for observations of interactions between two or more microbial species. This method enables the precise control of environmental conditions, various combinations of species ratios, and handling of large sample volumes for high-throughput parallel experiments. The kChip technology utilizes microfluidic chips to isolate and culture different microbial combinations in tiny droplets, gathering information on microbial interactions, deciphering community interaction mechanisms, screening natural products, and identifying functional microbiomes [101].

Furthermore, researchers have developed various co-culture tools based on semi-permeable membranes for studying cellular interactions under non-contact conditions. This technique is extensively utilized to explore the interactions involved in migration, invasion, polarity, and secretion among microorganisms, between microorganisms and host cells, and between cells. The Corning Transwell includes a nested membrane with micropores, which allows for material exchange between the upper and lower chambers without cell contact by controlling the size of the micropores [102]. Similarly, Moutinho modified this into a novel co-culture plate with a vertically oriented membrane, allowing the co-culture to be connected to a standard 96-well plate reader to monitor the optical density of the culture [103]. The BioMe plate is a redesigned microplate device where paired wells are separated by a porous membrane. The BioMe facilitates the measurement of dynamic microbial interactions, where each individual unit (well) can be set with different culturing conditions including nutrients, pH, temperature, and oxygen concentration to simulate various microenvironments. Equipped with advanced optical and electrochemical sensors, BioMe plates can monitor microbial growth dynamics, metabolic products, and environmental changes in real time [104].

### 4.3. Quantifying Interactions

Mathematical modeling is often used to more intuitively understand complex interactions [105]. Microbial community dynamics modeling based on theoretical ecology uses ordinary differential equations to describe the temporal evolution of microbial species abundances. The Lotka–Volterra model is the most widely used [106]. This model measures the impact of an individual from population A on the growth of an individual in population B using interaction coefficients. The Lotka–Volterra model has been applied in simulating interactions between yeasts and bacteria in cheese communities [107]. Due to the highly complex spatial structure of microbial communities, the interactions and richness of microbes change not only over time but also across space. In such cases, partial differential equation (PDE) models are commonly used to capture the system’s dynamics at different locations. The most widely used PDE model is the reaction–diffusion equation [108]. A previous study used an improved Monod model to describe the co-cultivation of *Phanerochaete chrysosporium* and *Schizophyllum commune* under SSF in fruit peels [109].

## 5. Interactions between AAB and Other Microorganisms

The interactions among microorganisms occur at the strain level, primarily mediated by different genotypes. In food fermentations involving AAB, other microbes such as yeasts, LAB, and *bacilli* strains are commonly present. The relationships among these microorganisms can vary depending on the nutritional environment and different stages of fermentation. This section will explore examples of microbial interactions in various environments.

### 5.1. AAB and LAB

LAB are diverse in fermented foods, primarily metabolizing carbohydrates to produce lactic acid. In these products, LAB can utilize multiple substrates, tolerate low pH environments, produce antimicrobial substances, impart acidity to food, and provide precursors for flavor synthesis. The interaction between AAB and LAB is one of the most studied among interactions with other microbes. Metagenomic data suggest that the relationship between AAB and LAB varies across different fermented foods. For example, in Colombian coffee bean fermentation, *Leuconostoc* shows a positive correlation with *Acetobacter*, although AAB has little correlation with the final sensory scores of the coffee [58]. In Nongxiangxing Daqu, LAB shows positive correlations with *Acetobacter* and *Gluconobacter* [110]. However, in Qula (a cheese-like product) from the Qinghai–Tibetan Plateau in China, *Acetobacter* and *Gluconobacter* are negatively correlated with *Lactobacillus*. Negative correlations between AAB and LAB are also commonly observed in grain and fruit vinegar fermentations, such as in Ziziphus jujuba vinegar, where *Acetobacter* shows negative correlations with most bacteria, while *Komagataeibacter* shows no correlation with any genera [111]. During the fermentation of Zhenjiang aromatic vinegar (0–9 days), *Acetobacter* primarily shows negative correlations with *Lactobacillus* and *Acetilactobacillus,* with interactions diminishing as fermentation progresses [112]. Different types of LAB and AAB also display varying interaction patterns. An in situ microbial network analysis of solid-state vinegar fermentation found that the primary microbes *Acetobacter pasteurianus* and *Lactobacillus helveticus* were negatively correlated, and *Acetobacter pasteurianus* was also negatively correlated with *Limosilactobacillus fermentum* and *Lactobacillus acetotolerans*, while positive correlations were observed with *Lactobacillus aceti* and *Lactiplantibacillus plantarum* [113]. In sourdough, although AAB presence was detected, gene amplicon data sets showed no significant correlations with other microbial groups [56].

Extensive research exists on the interaction mechanisms and relationships between specific strains of AAB and LAB. The interaction between *Acetobacter pasteurianus* and *Lactobacillus helveticus* in grain vinegar fermentation and in vitro simulated environments can be divided into two phases: from a positive correlation in the first phase to a negative correlation in the second phase [114]. Changes in interaction are primarily determined by the concentrations of ethanol, lactic acid, and particularly acetic acid [112,114]. Low lactic acid concentrations promote the growth and acetic acid production of *Acetobacter pasteurianus*, resulting in higher cell counts and faster acid production rates in co-culture than in solo cultures. However, acetic acid produced by *Acetobacter pasteurianus* has a significant inhibitory and lethal effect on *Lactobacillus helveticus* [112]. Transcriptomic analysis also confirmed that co-culturing had a greater transcriptional impact on *Lactobacillus helveticus* CGMCC 12062 than on *Acetobacter pasteurianus* [112]. Positive correlations between *Acetobacter* and *Lactobacillus* at low acetic acid concentrations were also observed. Using the newly developed BioMe plate, interactions between *Acetobacter oryzifermentans*, *Levilactobacillus brevis*, and *Lactiplantibacillus plantarum* in the gut microbiome of *Drosophila melanogaster* were observed. Metabolites from *Lactobacillus* in non-contact co-culture promoted the growth of *Acetobacter oryzifermentans* without affecting *Lactobacillus*, demonstrating a unidirectional symbiotic relationship rather than a mutually interactive one [104].

Incorporating *Gluconacetobacter* spp. isolated from apple vinegar into milk kefir grains aids in the formation of exopolysaccharides and increases the biomass of kefir grains without negatively affecting the sensory properties of kefir or the microbial community, which includes *Lactobacillus* spp., *Lactococcus* spp., yeasts, *Lactobacillus acidophilus*, and *Bifidobacterium* spp. [115]. Research has found a symbiotic relationship between LAB isolated from kefir (*Lactobacillus* sp., *Lactococcus lactis*, and *Leuconostoc* sp.) and *Gluconobacter* spp. The LAB appear to play a significant role in the survival and activity of *Gluconobacter* sp. A4. Metabolites from LAB, such as xylitol and acetic acid, are utilized by *Gluconobacter* sp. A4, promoting its growth and the production of D-saccharic acid 1,4-lactone [116].

### 5.2. AAB and Yeasts

Yeast species are diverse, with varied metabolic types that can produce substances such as ethanol and free amino acids, which are beneficial for the growth of AAB. However, the acetic acid produced by AAB has an inhibitory effect on yeast. Thus, the relationship between AAB and yeasts in fermented foods is often observed as a commensal symbiosis. In the fermentation of Xiaoqu light-aroma Baijiu, AAB can influence the succession of fungi, especially non-brewing yeasts [117]. Negative correlations were found between *Pichia* and *Wickerhamomyces* with *Acetobacter*, as well as between *Saccharomyces* and *Gluconobacter*, and between *Kazachstania* and *Gluconobacter* [117]. The Lotka–Volterra model was used to assess yeast–yeast interactions and yeast–bacteria interactions during the ripening of smeared cheese. The study found negative interactions among yeasts, where yeasts were key species for bacterial development, and bacterial–bacterial interactions could not be clearly identified [107]. *Acetobacter pasteurianus* can increase the content of polyphenols in kombucha, specifically increasing the content of epigallocatechin gallate. *Acetobacter pasteurianus* is a strong competitor in the kombucha environment, capable of reducing the number of live yeast cells by producing more acid [118].

In kombucha fermentation using sucrose as a carbon source, Tran et al. studied the interactions between yeasts (*Brettanomyces bruxellensis*, *Hanseniaspora valbyensis*, *Saccharomyces cerevisiae*) and AAB (*Acetobacter indonesiensis*, *Acetobacter papayae*, and *Komagataeibacter saccharivorans*) through pure and co-cultivation. An analysis of the changes in sugars, ethanol, organic acids, free amino acids, and viable cell counts after fermentation revealed that AAB can grow independently of substrates provided by yeasts, but the presence of AAB in co-cultures reduced the number of yeast cells, indicating a non-strict parasitic relationship between yeasts and AAB. Besides glucose, fructose, and ethanol, yeasts can also release nitrogenous substrates by converting bound amino acids (such as proteins and peptides) into free amino acids. Yeast metabolism directly influences the flux of substrates available to AAB, thus regulating AAB metabolism. Conversely, AAB induces brewing yeasts to produce substrates through indirect changes in its metabolism, enhancing the benefits of this commensal relationship. Yeasts and AAB that cannot effectively hydrolyze sucrose rely on yeasts with high enzymatic activity to access released monosaccharides; these are considered “cheaters” in the original microbial community [119].

### 5.3. AAB and bacilli

Bacilli, as aerobic, spore-producing, heat-resistant, rod-shaped bacteria, are widespread in the production of fermented foods. They possess the ability to hydrolyze proteins and starches and metabolize various flavor compounds. One of the most notable interactions between AAB and bacilli is in the fermentation production of vitamin C. Commonly, *Gluconobacter oxydans* and *Bacillus megaterium* are used to convert L-sorbose to 2-keto-L-gulonic acid (2-KGA), with *Gluconobacter oxydans* being the 2-KGA-producing strain. When cultured alone, *Gluconobacter oxydans* grows slowly and produces acid in limited quantities. *Bacillus megaterium* acts as a companion strain, stimulating the growth of *Gluconobacter oxydans* or the production of 2-KGA by *Gluconobacter oxydans* [120]. The generalized Lotka–Volterra equations describing the mixed growth of *Gluconobacter oxydans* and *Bacillus megaterium* suggest that the interaction between them is predatory, with *Gluconobacter oxydans* as the predator and *Bacillus megaterium* as its prey [121]. During the fermentation of Zhenjiang aromatic vinegar, AAB and *Bacillus* show a positive correlation [122]. In the solid-state fermentation process of sauce-flavored Baijiu, *Bacillus* are negatively correlated with AAB [123]. In the fermentation of highland barley wine, AAB, as contributors to flavor, show weak negative correlations with almost all other bacterial genera, including *Bacillus subtilis* [124]. In various fermented foods, inoculating with bacilli, which possess a rich enzymatic system, helps break down large organic molecules into smaller molecules, enhancing sulfur metabolism, affecting microbial community structure, and ultimately influencing the content of flavor compounds [125,126,127].

### 5.4. The Impact of AAB on Other Microorganisms

In the solid-state fermentation of Chinese grain vinegar, it was found that non-dominant microbial communities play a crucial role in network stability. Research has shown that *Komagataeibacter europaeus* is the most coexistent non-abundant species, playing a significant role in the functionality and resilience of the microbial community. When *Komagataeibacter europaeus* JNP1 was bioaugmented, it was found to regulate the composition of the microbial group and enhance the efficiency of biological processes by increasing the acetic acid content and decreasing reducing sugars. Additionally, *Komagataeibacter europaeus* has demonstrated the ability to impart stability to both primary and secondary abundant members within the microbial community [128].

## 6. Molecular Mechanisms of Interactions between AAB and Other Microorganisms

The interactions between microorganisms are mediated through various molecular and physiological mechanisms. A comprehensive consideration of the scope and intensity of interactions, along with the community environment, can enhance our understanding of the molecular mechanisms of bacterial interactions.

### 6.1. Metabolites

Microbial interactions regularly influence the growth and metabolism of other microorganisms via alterations to the environment brought about by metabolites. For fermented food, the metabolic complementarity contributes to community stability and the efficient realization of specific functions. This metabolic complementarity is most evident in the provision and release of nutrients essential for microbial growth in the surrounding environment. For instance, the fermentation supernatant of *Lactobacillus helveticus*, containing extracellular aminopeptidases, can hydrolyze to produce more free amino acids, promoting the growth metabolism of *Acetobacter pasteurianus* and increasing the acid production rate. The metabolic products of one microorganism promote the growth and survival of others. For example, studies on single and co-cultures of *Acetobacter pasteurianus* with different LAB (*Limosilactobacillus reuteri*, *Lentilactobacillus buchneri*, *Levilactobacillus brevis*, *Limosilactobacillus fermentum*, *Lactobacillus casei*, *Lactiplantibacillus plantarum*) in grain vinegar and apple vinegar fermentations or simulations found that *Acetobacter pasteurianus* prioritizes ethanol before glucose, while lactobacilli prioritize glucose before ethanol [129]. The growth of *Acetobacter pasteurianus* is almost unaffected, while the growth of LAB is significantly inhibited, likely due to intolerance to the produced acetic acid. LAB contributed to the accumulation of free amino acids and lactic acid. During co-cultivation, *Acetobacter* can incompletely oxidize lactic acid, aiding in the accumulation of acetoin and improving the quality of fermented vinegar [130]. A similar process was observed in cocoa bean fermentation, where the final products were acetic acid and ethyl acetate. However, different species of AAB (*Acetobacter pasteurianus*, *Acetobacter ghanensis*, *Acetobacter senegalensis*, and *Acetobacter fabarum*) differ in their rates of lactic acid utilization and oxidation to ethyl acetate. Additionally, the metabolic products of different AAB vary, with *Acetobacter pasteurianus* 386B and *Acetobacter ghanensis* LMG 23848 capable of oxidizing mannitol into fructose [131]. *Acetobacter pasteurianus* was co-fermented with *Lacticaseibacillus casei*, *Lactobacillus helveticus*, and *Lactiplantibacillus plantarum* in combinations in Jujube Puree, where different combinations affected the content of free amino acids, phenolic compounds, and volatile flavor compounds after fermentation [132]. Further, in kombucha, the interactions between two yeast species (*Brettanomyces bruxellensis* and *Hanseniaspora valbyensis*) and an acetic acid bacterium (*Acetobacter indonesiensis*) were studied from a metabolomic perspective. The yeast–AAB interactions varied depending on the yeast species, significantly affecting compounds from different metabolic pathways. In co-cultures with *Brettanomyces bruxellensis*, there was an increase in fatty acids and peptides through the consumption of sucrose, fatty acids, and polysaccharides. When co-cultured with *Hanseniaspora valbyensis*, phenylpyruvate and a few other compounds were produced through the consumption of polyphenols, peptides, fatty acids, phenolic acids, and putative isopropyl malate. Notably, hydroxystearic acid produced by yeasts in kombucha was utilized and converted by co-cultured *Acetobacter indonesiensis* into γ-dodecalactone, enhancing the product’s flavor [133].

### 6.2. Oxidative Stress

AAB, through their unique oxidative fermentation mechanism, can efficiently transfer electrons and generate energy via specialized respiratory chains on their cell membranes, which increases the production of reactive oxygen species (ROS) in the environment, such as superoxide anions, hydrogen peroxide, and hydroxyl radicals [134]. These ROS can damage cellular structures and molecules, such as lipids, proteins, and DNA, thereby impacting coexisting microorganisms [135]. The ROS produced by AAB may directly inhibit or kill sensitive microbes, especially those with weaker antioxidant defenses. This inhibitory effect can reduce competitors, helping AAB to dominate microbial communities. In microbes, sustained oxidative stress can induce antioxidant responses, such as increasing the expression of antioxidant enzymes (superoxide dismutase, catalase, and peroxidase) and the synthesis of other antioxidant molecules [136]. This adaptive response helps microbes survive and thrive in oxidative environments. Microbial cells have a complex antioxidant defense system, including various antioxidant enzymes (superoxide dismutase (SOD), catalase (CAT), glutathione peroxidase (GPx) and non-enzymatic antioxidants (glutathione (GSH), vitamin E, vitamin C). These antioxidant systems can scavenge or neutralize ROS, protecting the cells from oxidative damage. AAB and their oxidative fermentation mechanisms may play a broader role in microbial ecosystems, influencing the redox balance in the environment and thus affecting the ecological status and metabolic activity of other microbes [18].

### 6.3. Gene Expression Regulation

In microbial interactions, the regulation of gene expression plays a crucial role. Gene expression regulation involves transcriptional regulation [137], epigenetic control [137], post-transcriptional regulation [138], protein modification and degradation [139], and horizontal gene transfer, all of which respond to the mediating molecules or environmental factors influencing microbial interactions.

A transcriptomic analysis of *Lactobacillus helveticus* and *Acetobacter pasteurianus* co-cultured during the simulated fermentation process of Shanxi aged vinegar revealed that in *Lactobacillus helveticus*, genes involved in the metabolic pathways of starch and sucrose, galactose, fatty acids, and some amino acids were down-regulated, while genes involved in glycerophospholipid metabolism, energy (ABC transporters), pyrimidine, and purine pathways were up-regulated. In *Acetobacter pasteurianus*, genes for the KEGG pathways of sugar, amino acid, purine, and pyrimidine metabolism were up-regulated under co-culture conditions, while the metabolic pathways of pyruvate, glyoxylate, fructose, and mannose were down-regulated compared to its mono-culture condition. These results suggest an amensalism phenomenon in the co-culture of *Acetobacter pasteurianus* and *Lactobacillus helveticus* [140]. A co-culture technique applied to *Komagataeibacter xylinum* and *Lactococcus lactis* subsp. *lactis* successfully produced bacterial cellulose/lactic bacterium peptide films with improved antimicrobial activity and mechanical properties. The increase in lactic bacterium peptide production was associated with up-regulated gene expression, demonstrating that the increase in secondary metabolites following microbial interactions is regulated by gene expression [141].

In genomic analyses and co-culture studies of AAB, horizontal gene transfer in AAB is often observed. Comparative genomic analyses have shown that species such as *Acetobacter pasteurianus*, *Acetobacter ghanensis*, *Acetobacter senegalensis*, *Komagataeibacter medellinensis*, and *Komagataeibacter nataicola* possess multiple genes acquired through horizontal gene transfer, enhancing their adaptability and impacting metabolic processes [142,143]. This study has revealed that *Acetobacter* and *Komagataeibacter* can acquire antibiotic resistance genes through horizontal gene transfer mechanisms such as conjugation, transformation, or transduction, giving them a competitive edge against other bacteria. *Gluconobacter oxydans*, in the production of vitamin C, has shown the ability to acquire new metabolic pathways through horizontal gene transfer [144].

### 6.4. Signal Transduction

Quorum sensing is a communication mechanism among bacteria that regulates collective behavior through the synthesis and secretion of signaling molecules [145]. In AAB, this system significantly impacts acid production and bacterial cellulose synthesis, playing a crucial role in interactions with other microorganisms [146,147,148]. In AAB, quorum sensing signaling molecules are primarily AHLs (acyl-homoserine lactones), which have been detected in *Komagataeibacter*, *Gluconacetobacter*, and *Acetobacter* [146,149,150]. In *Komagataeibacter*, gene modules related to quorum sensing have been identified, which enhance the microbial system’s tolerance to acetic acid and are also associated with biofilm formation [151]. The genome of *Acetobacter pasteurianus* was subjected to bioinformatics prediction, which revealed the absence of genes encoding AHLs and AI-2 signaling molecules [152]. However, the presence of AHLs and a negative correlation with acetic acid fermentation were detected in the culture broth [146]. The number of *LuxI* and *LuxR* homolog sequences varies among AAB genomes isolated from different sources, which likely relates to the specific growth environments of the strains [146]. *Komagataeibacter europaeus* shows the most connections with other microbes in grain vinegar fermentation, possibly due to its quorum sensing effects [128].

Outer membrane vesicles (OMVs) are spherical nanostructures naturally released from the outer membrane of bacteria, primarily found in Gram-negative bacteria [153]. These vesicles are rich in lipopolysaccharides, phospholipids, proteins, and nucleic acids. OMVs can fuse with the membranes of other bacterial cells, releasing their contents into the recipient cells and thereby affecting the function and behavior of these cells. In Gram-negative bacteria, such as *Escherichia coli* and *Pseudomonas aeruginosa* [154,155], OMVs have been found to carry toxins, enzymes, and other molecules that influence the bacteria’s survival strategies and interactions with hosts. Although there is not yet widespread reports of detailed studies on OMVs in AAB (Gram-negative bacteria), this is a potential research area that may reveal new functions and applications for these economically important bacteria in the future.

## 7. Conclusions and Perspectives

A variety of AAB species such as *Acetobacter* spp., *Gluconobacter* spp., and *Komagataeibacter* spp. participate in food fermentation processes including those for vinegar, sour beer, fermented cocoa and coffee beans, kefir beverages, kombucha, and sourdough. In these fermented foods, they often coexist with yeasts, LAB, and bacilli, demonstrating certain interactions. The interactions between particular pairs of species dynamically change in different fermentation systems and at different fermentation stages. It has been observed that other microbes predominantly facilitate AAB growth or acid production through nutrient supply, oxidative stress, gene expression regulation, and signal transduction. In co-cultivation, AAB primarily exhibit an inhibitory effect on yeasts, LAB, and bacilli, mainly through the production of acetic acid, which inhibits the growth of other microbes. The production of ROS, acquisition of horizontal transfer genes, quorum sensing signal degradation, and secretion of OMVs also provide AAB with a competitive advantage within the microbial community. These various microbial interactions lead to community succession and distinguish the final products of further fermentation. Consequently, we can utilize the known interactions of AAB with other microbes under specific conditions to design and optimize the fermentation process. For example, the positive interaction between LAB and AAB can be utilized to design synthetic communities for enhancing the flavor and yield of vinegar.

Thanks to the rapid development of sequencing technologies, we have obtained extensive information on microbial interactions within the microbial networks in fermented foods. However, from existing research, there is a lack of in-depth explanation of the molecular mechanisms of interactions between AAB and other microbes, especially how they communicate chemically, what signals are exchanged, and how metabolic complementarity or competitive relationships affect each other. This includes studies on the survival strategies and ecological niche relationships between microbes. Furthermore, standardized methods for studying microbial interactions, particularly those involving more than three species, are yet to be established. These research directions will help deepen our understanding of the ecological functions and application potential of AAB, providing new insights and technologies for the fields of food industry and biotechnology.

## Figures and Tables

**Table 1:** Common species of acetic acid bacteria found in fermented foods.

Food Category	Fermented Product	AAB Species	References
Vinegars	Wine vinegar	*Acetobacter aceti*, *Acetobacter cerevisiae*, *Acetobacter malorum*, *Acetobacter pasteurianus*, *Gluconacetobacter intermedius*, *Komagataeibacter europaeus*	[32]
	Wine vinegar	*Komagataeibacter europaeus*	[33]
	Rice vinegar (Komesu and Kurosu)	*Acetobacter pasteurianus*	[34]
	Italy traditional balsamic vinegar	*Acetobacter aceti*, *Acetobacter pasteurianus*, *Komagataeibacter xylinus*	[35]
	Turkey traditional apple vinegar	*Acetobacter okinawensis*, *Komagataeibacter europaeus*, *Komagataeibacter hansenii*, *Komagataeibacter xylinus*	[36]
	Turkey traditional grape vinegar	*Acetobacter indonesiensis*, *Komagataeibacter europaeus*, *Komagataeibacter hansenii*, *Komagataeibacter xylinus*	[36]
	Apple vinegar	*Acetobacter hansenii*, *Acetobacter pasteurianus*, *Komagataeibacter europaeus*, *Komagataeibacter xylinus*	[33]
	Estamaran date vinegar	*Komagataeibacter xylinus*	[37]
	Sichuan Baoning vinegar	*Acetobacter cibinongens*, *Acetobacter ghanensis*, *Acetobacter pasteurianus*, *Acetobacter pomorum*	[38]
	Zhenjiang vinegar	*Acetobacter aceti*, *Acetobacter pasteurianus*	[39]
	Shanxi aged vinegar	*Acetobacter indonesiensis*, *Acetobacter malorum*, *Acetobacter orientalis*, *Acetobacter pasteurianus*, *Acetobacter senegalensis*, *Gluconobacter oxydans*	[40]
	Korea grain vinegar	*Acetobacter ghanensis*	[41]
Beverages	Acidic beers	*Acetobacter lambici*, *Acetobacter malorum*, *Acetobacter orientalis*, *Acetobacter pasteurianus*, *Gluconobacter cerevisiae*	[42,43,44,45,46]
	Water kefir	*Acetobacter sicerae*	[47]
	Milk kefir	*Acetobacter fabarum*	[48]
	Kombucha	*Komagataeibacter xylinus*	[49]
	Kombucha	*Acetobacter ascendens*, *Acetobacter cibinongensis*, *Acetobacter malorum*, *Acetobacter orientalis*, *Acetobacter pasteurianus*, *Acetobacter pomorum*, *Gluconobacter oxydans*	[50]
	Singapore kombucha	*Komagataeibacter rhaeticus*, *Komagataeibacter saccharivorans*, *Komagataeibacter xylinus*	[51]
	Malt-fermented soft drinks	*Gluconobacter oxydans*	[52]
	Post-alcoholic fermentation wines	*Gluconobacter* sp., *Acetobacter* sp.,	[53]
Starters	Luxiang-flavor Jiupei	*Acetobacter*	[54]
	Wheat sourdoughs	*Acetobacter pasteurianus*, *Gluconobacter oxydans*	[55]
	Sourdough starter	*Acetobacter lovaniensis*, *Acetobacter malorum*, *Acetobacter pasteurianus*, *Acetobacter tropicalis*, *Gluconobacter frateurii*, *Gluconobacter sphaericus*, *Komagataeibacter*,	[56]
Others	Cocoa	*Acetobacter ghanensis*, *Acetobacter malorum*, *Acetobacter okinawensis*, *Acetobacter pasteurianus*, *Acetobacter tropicalis*, *Gluconobacter oxydans*	[57]
	Coffee fermentation	*Acetobacter* sp., *Gluconobacter* sp.	[58,59,60,61,62]
	Nata de coco	*Komagataeibacter nataicola*, *Komagataeibacter* sp.	[63,64,65]
	Acidic gruel	*Acetobacter*	[66]

## Data Availability

No new data were created or analyzed in this study. Data sharing is not applicable to this article.
